# Peak myocardial work assessment to detect coronary ischemia during dobutamine stress echocardiography

**DOI:** 10.3389/fcvm.2025.1556991

**Published:** 2025-04-08

**Authors:** Salima Qamruddin, Chen Fang, Sergey Kachur, Sahil Bharwani, Andrew Elagizi, Merrill Stewart, Daniel P. Morin, Otto A. Smiseth, Yvonne E. Gilliland

**Affiliations:** ^1^ Division of Cardiovascular Disease, John Ochsner Heart and Vascular Institute, New Orleans, LA, United States; ^2^ Ochsner Clinical School, The University of Queensland School of Medicine, New Orleans, LA, United States; ^3^ Echocardiography Laboratory, Ochsner Medical Center, New Orleans, LA, United States; ^4^ Division of Cardiovascular and Pulmonary Diseases, Oslo University Hospital, Rikshospitalet and University of Oslo, Oslo, Norway

**Keywords:** dobutamine stress echocardiography, global longitudinal strain, global myocardial work index, global myocardial work efficiency, pressure strain loop

## Abstract

**Introduction:**

Peak global myocardial work efficiency (GWE), a measure of peak global myocardial constructive to wasted work ratio, has been shown to discriminate coronary ischemia during treadmill stress echocardiography (SE). We wanted to assess additive utility of peak global longitudinal strain (GLS), global work index (GWI), and GWE in improving positive predictive value (PPV) of an abnormal dobutamine stress echocardiography (DSE) and calculate cost-savings by avoiding secondary tests.

**Methods:**

We prospectively enrolled patients with abnormal DSE who underwent secondary confirmatory tests to confirm significant CAD as our primary cohort, and measured baseline and peak GLS, GWI, and GWE. We also included a control group with normal DSE results and similar measurements. The cost of secondary testing was used to calculate potential savings.

**Results:**

Among the 45 patients (71% females, mean age 60 ± 12 yrs.), 9 had significant CAD, 11 had non-significant CAD, and 25 were controls (N). Patients with significant CAD had significantly lower peak GLS [−15 (−17, −12.5) vs. −20 (−22, −19.5)%, *p* < 0.001], peak GWI [1,057 (810.5, 1,057) vs. 2,245 (1,928.5, 2,961) mmHg%, *p* = 0.02], peak GWE [82 (74.5, 86.5) vs. 89 [(86, 93.5)%, *p* = 0.001], and peak GCW [1,618 (1,153.5, 2,003) vs. 2,585 (2,262.5, 3,262) mmHg%, *p* = 0.02] compared to control. ROC analysis demonstrated peak GWE [AUC 0.76 (0.55, 0.97) *p* = 0.01] to discriminate coronary ischemia. Incorporating peak GWE of <87% into abnormal DSE interpretation improved PPV from 45% to 81%, resulting in an estimated cost savings of $8,274.00 per screened patient.

**Conclusions:**

Incorporating peak GWE into standard DSE interpretation enhanced diagnostic accuracy and reduced the cost of downstream testing.

## 1 Introduction

Early and accurate detection of myocardial ischemia due to obstructive coronary artery disease (CAD) is important to avoid potentially life-threatening complications. Stress echocardiography (SE) is an initial non-invasive diagnostic test recommended for patients suspected of having CAD. Likewise, Dobutamine stress echocardiography (DSE) is a validated test for diagnosing ischemia in older patients and those who cannot exercise on a treadmill (1). Acquiring additional objective non-invasive parameters to improve diagnostic accuracy of stress testing and detect ischemia beyond wall motion assessment has been proposed (2).

**Figure 1:**
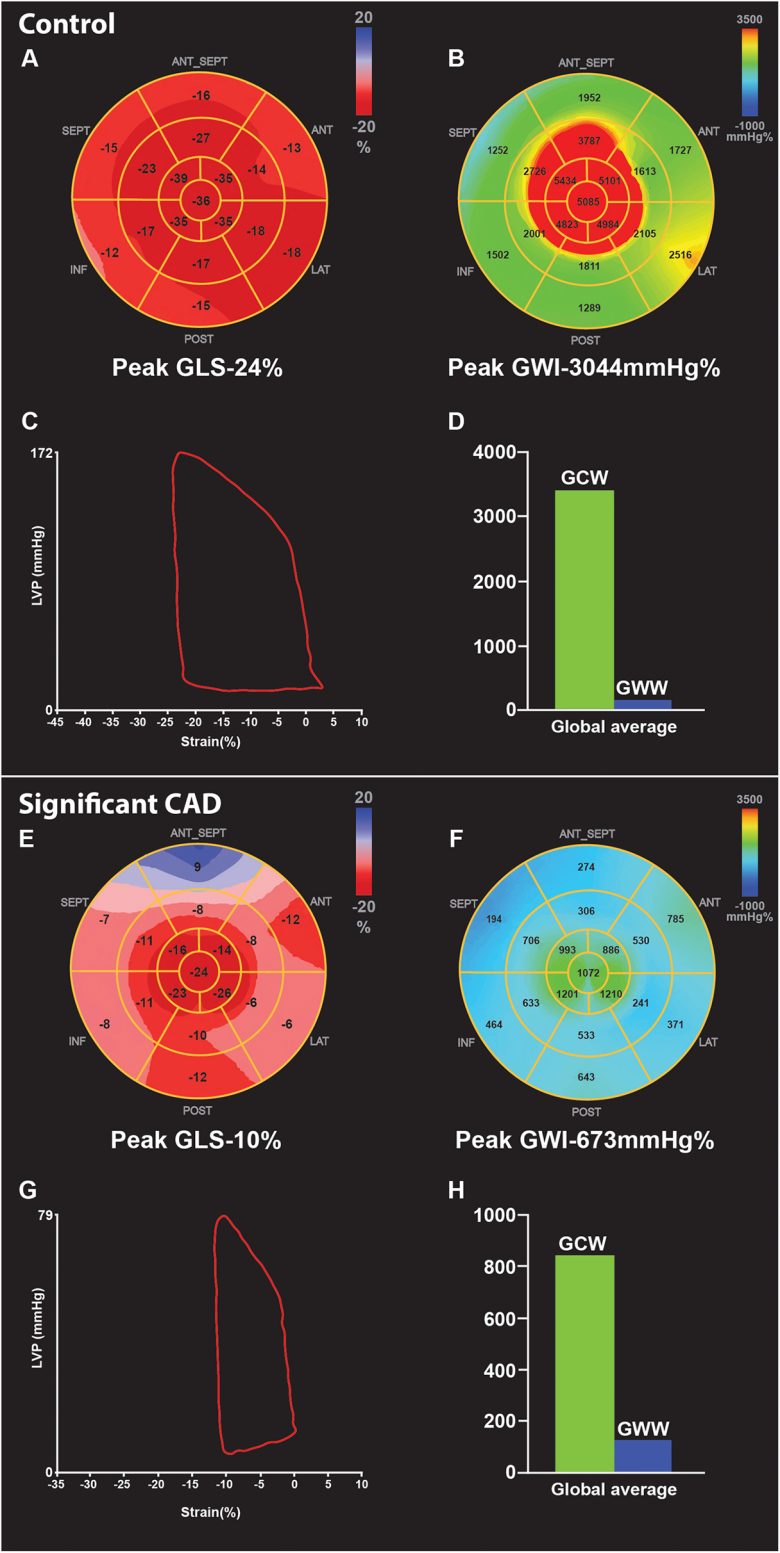
Example of GLS and myocardial work parameters at peak stress in two patients undergoing DSE. First is a control patient with normal DSE (A-D), showing increase in GLS [rest −21% to peak −24% **(A)**], and GWI [rest 2,031 mmHg% to peak 3,044 mmHg% **(B)**] compared to rest. Pressure strain loop (PSL) shows adequate GWI- area under the curve **(C)**, and the bar graph showing high GCW and minimal GWW**(D)** at peak. In comparison, patient with significant CAD shows reduced peak GLS [rest −15% to peak −10% **(E)**], and reduced GWI [rest 1,014 mmHg% to peak 673 mmHg% **(F)**] compare to rest, due to obstructive lesion in the mid LAD and distal LCX confirmed on coronary angiography, PSL loop also shows reduced GWI- area under the curve **(G)**, compared to normal DSE PSL **(C)** Bar graph **(H)** shows reduced GCW and higher GWW in significant CAD compared to **(D)** control. CAD, coronary artery disease; DSE, dobutamine stress echocardiogram; GLS, global longitudinal strain; GWI, global myocardial work index; GCW, global myocardial constructive work; GWW, global myocardial wasted work; PSL, pressure strain loop.

Two-dimensional echocardiography with speckle tracking has been validated and is an accepted technique to measure the strain (deformation) of the left ventricular (LV) wall (3). Global longitudinal strain (GLS) measures deformation in the subendocardial myocardium, which is susceptible to early ischemic changes. It precedes visual detection of regional wall motion abnormalities, and any changes in left ventricular ejection fraction (LVEF), which are influenced by altered myocardial thickening in the radial direction. Therefore, GLS is capable of early detection of subclinical regional and global left ventricular contractile dysfunction due to myocardial ischemia (4–7).

Since GLS is sensitive to afterload limiting its accuracy, global myocardial work index (GWI), a novel echocardiographic parameter, was designed to resolve whether GLS reduction is due to reduced contractility (reflected as reduced myocardial work), or increased afterload (reflected as increased myocardial work). This correction is performed by using systolic blood pressure (SBP) as a non-invasive surrogate for left ventricular systolic blood pressure (8, 9). The current method for assessment of myocardial work was validated with myocardial glucose metabolism and invasive left ventricular pressure measurements (10). Normal ranges of myocardial work (MW) have been established (11, 12). MW indices measure the area under the pressure-strain loop (PSL) constructed from non-invasively measured LV pressure curves and LV longitudinal strain. Global myocardial work index (GWI), calculated as the average of all segmental values, represents the total work within the area of the PSL during the time from mitral valve closure (MVC) to mitral valve opening (MVO). Global myocardial work efficiency (GWE) is a measure of global myocardial constructive myocardial work (GCW) vs. global myocardial wasted myocardial work (GWW). Resting GWI has been shown to be superior to resting GLS in detecting significant CAD and acute coronary occlusion in those referred for chest pain (13, 14). Peak GLS and peak GWE has also been shown to discriminate coronary ischemia better than peak wall motion in those undergoing treadmill echocardiography (15).

The primary hypothesis of our study was that adding the above-stated myocardial work parameters at peak stress may further improve the diagnostic accuracy of an abnormal DSE when added to the standard wall motion assessment, given the high false positive (FP) rate (28%) in SE (16). The secondary hypothesis was that improving the diagnostic accuracy of DSE by adding these parameters may result in cost savings by avoiding downstream testing.

The primary objective was to assess the utility of peak GLS, GWI, and GWE in improving positive predictive value (PPV) of an abnormal DSE. The secondary objectives were to compare these values to, a) a group of control patients, and b) to calculate potential cost-savings from avoiding secondary testing.

## 2 Materials and methods

### 2.1 Study population

The study was approved by the Institutional Review Board review at Ochsner Medical Center. We sequentially enrolled patients undergoing DSE at Ochsner Medical Center from 12/2019-12/2021. We included patients with interpretable apical four, two, and three chamber views at rest and peak DSE with adequate frame rate [50–80 frames per sec (fps)]. Patients with resting wall motion abnormalities, prior LV dysfunction (LVEF < 50%), valvular disease, hypertrophic cardiomyopathy, presence of left ventricular outflow tract obstruction, atrial fibrillation, left bundle branch block, paced rhythm, and use of contrast agents were excluded.

### 2.2 Dobutamine stress echocardiography

All studies were performed on the Vivid E95 ver. 203 (GE HealthCare, Chicago, IL). Baseline patient echocardiographic characteristics were extracted from electronic health record EPIC (Verona, WI) and CUPID, EPIC (Verona, WI) respectively. All images were acquired by a standard DSE protocol recommended by the American Society of Echocardiography (ASE) (1). Systolic wall thickening and wall motion score index (WMSI) were assessed in a 17-segment LV model according to a four-point score at rest and at stress (1, normal; 2, hypokinetic; 3, akinetic; 4, dyskinetic motion). WMSI was calculated by dividing the sum of segmental values by the number of visualized segments (17).

### 2.3 Secondary confirmatory testing (coronary angiography, positron emission tomography, single photon-emission computed tomography)

Determination by visual assessment of significant CAD on coronary angiography was defined as >75% luminal stenosis in one or more major epicardial vessels, or >50% in the left main coronary artery. Coronary ischemia by positron emission tomography (PET) was defined as a stress-induced perfusion defect in greater than 10% of the LV myocardium with a relative radiotracer uptake of less than 60% of maximum (18). Coronary ischemia by single photon-emission computed tomography (SPECT) was defined semi-quantitatively using a summed difference score of 4 or greater in at least two contiguous segments, as evaluated by two independent cardiologists. Semi-quantitative rest, stress, and difference scores were calculated in accordance with the most recently published American Society of Nuclear Cardiology guidelines on SPECT interpretation (19). Rest and DSE images were independently interpreted by two experienced echocardiographers. The images from coronary angiograms, PETs, and SPECTs were interpreted by an experienced interventional cardiologist and nuclear cardiologist respectively, who were blinded to the echocardiographic results. The secondary tests were performed within 24 ± 23 days after abnormal DSE.

Patients were labeled to have significant CAD if they had abnormal DSE (WMSI >1) and were found to have significant CAD as defined above, correlating with the area of echocardiographic ischemia. Patients were deemed to have non-significant CAD if they had an abnormal DSE (WMSI >1), but the secondary test indicated absence of coronary ischemia. The control group (N) included patients with normal DSE (WMSI <1) without secondary testing. However, these patients had a history of hypertension and hyperlipidemia, but no Diabetes Mellitus (DM), chronic kidney disease (CKD), or prior diagnosis of CAD. Hence, this was not a healthy cohort.

### 2.4 Myocardial work analysis

Standard DSE protocol images were obtained as recommended by ASE (1, 2). Aortic valve and mitral valve opening and closure timings were measured at rest using pulse wave Doppler at the left ventricular outflow tract and mitral valve (MV) leaflets, respectively. Systolic brachial blood pressure was obtained in the supine position at rest and during each stage of the DSE protocol. All data were acquired using the GE automated function imaging (AFI) stress protocol. All calculations were performed offline. The 17-segment LV GLS model was computed with automated function imaging (AFI) software. Baseline and Peak GWI were auto-calculated by the software after adding systolic blood pressure to calculated GLS. Segmental values of myocardial work (MW) were displayed in bull's-eye plots.

Global myocardial constructive work (GCW), which is work performed by all segments during shortening in systole, adding negative work during lengthening in isovolumic relaxation time (IVR), and Global myocardial wasted work (GWW), which is work performed by all segments during lengthening during systole, adding work performed during shortening in IVR, were displayed. Global myocardial work efficiency (GWE), which is GCW divided by the sum of GCW and GWW, was also auto calculated. GWI PSL were checked for accuracy before the data was accepted. All data were analyzed by a single physician with expertise in cardiac imaging (SQ). Intra-observer variability of GLS, GWI, and GWE recorded in 5 patients was 2.7%, 0.06%, and 1.4%, respectively.

### 2.5 Cost evaluation

Costs of secondary tests were calculated based on the mean cost of each follow-up test at our facility. An average cost savings per patient was derived by summing costs of secondary tests in all patients with an abnormal DSE and dividing it by the number of patients who required follow-up studies. In our facility, the average cost of each secondary study was: $12,600 for a coronary angiogram, $4,377 for a cardiac PET/CT with myocardial blood flow, and $1,356 for a SPECT stress test.

### 2.6 Statistical analysis

Statistical analysis was done using SPSS Version 30 (IBM Corporation, Chicago, IL). Descriptive statistics were generated for the study groups, whose distribution was found to be non-parametric. A Kruskal–Wallis's test was applied to compare groups, followed by Dunn's pairwise tests with Bonferroni correction for individual group comparison. Receiver operating characteristics (ROC) curves were created to identify the cutoff for the myocardial work parameters with the greatest change in improving diagnostic accuracy of an abnormal DSE. This point was chosen using Youden's index. PPV was calculated as a ratio of patients with significant CAD divided by all patients with abnormal DSE.

## 3 Results

### 3.1 Demographic data

55 patients met inclusion criteria. 10 patients were excluded: a) due to poor endocardial definition (*n* = 7), and b) lack of adequate apical three chamber view (*n* = 3). Of the remaining 45 patients, 20 had abnormal DSE, and 25 were enrolled as controls. Out of the 20 abnormal DSE patients, 9 had significant CAD, and 11 had non-significant CAD on secondary testing. Baseline characteristics of the population are summarized in Table 1. Females predominated in all three groups. Patients found to have significant CAD on secondary testing were older (66 ± 8 vs. 54 ± 11 yrs., *p* = 0.003), had higher prevalence of DM (43 vs. 0%, *p* = 0.04), and had prior history of CAD (86 vs. 20%, *p* = 0.001) compared to those with non-significant CAD. The average LVEF was 58 ± 15% with no significant difference between the groups (*p* = 0.35). HTN was more prevalent in the significant CAD group than in the non-significant CAD group (86 ± 38 vs. 66 ± 50%, *p* = 0.02), but less than in the control group (86 ± 38 vs. 93 ± 25%, *p* = 0.02). Baseline and peak heart rate, as well as systolic blood pressure, did not significantly differ between the groups (Table 1). The primary indications for obtaining stress echocardiography included chest pain, shortness of breath, dyspnea on exertion, and preoperative evaluation. 96% of patients had normal sinus rhythm (NSR), while 4% had NSR with right bundle branch block (RBBB) on EKG.

**Table 1:** Baseline clinical and echocardiographic characteristics of patients undergoing DSE.

Variables	Significant CAD	Non-significant CAD	Control	Adjusted *p*-values
	(*n* = 9)	(*n* = 11)	(*n* = 25)	
Patient characteristics				
Females (%)	71	81	69	0.7
Age (yrs.)	66 ± 8	54 ± 11	60 ± 12	0.003
HTN (%)	86	66	93	0.02
DM (%)	43	0	0	0.04
CAD (%)	86	20	0	0.001
CKD (%)	43	40	0	0.95
Baseline/Peak heart rate (bpm)	68 ± 11/126 ± 9	71 ± 12/130 ± 14	68 ± 13/133 ± 12	0.78/0.17
Baseline/Peak SBP (mmHg)	136 ± 46/146 ± 59	126 ± 18/170 ± 42	137 ± 14/155 ± 40	0.22/0.34
Echocardiographic findings				
LVEF (%)	62 ± 4	55 ± 20	58 ± 15	0.35
Baseline/Peak WMSI	1 ± 0/1.29 ± .13	1 ± 0/1.2 ± 0.12	1 ± 0/1 ± 0	0.4
Baseline GLS (%)	−18 [−20.5, −17.5]	−19 [−21, −17]	−20 [−22, −18]	0.51
Baseline GWI (mmHg%)	2,092 [1,806, 2,668.5]	1,867 [1,822, 2,171]	2,201 [1,936.5, 2,507]	0.18
Baseline GWE (%)	94 [89, 95.5]	91 [87, 95]	95 [92.5, 96.5]	0.12
Baseline GCW (mmHg%)	2,535 [2,205, 3,189]	2,395 [1,740, 2,936.5]	2,400 [2,076, 2,831.5]	0.58
Baseline GWW (mmHg%)	187 [93.5, 256.5]	167 [86, 443]	105 [67.5, 171]	0.069
Peak GLS (%)	−15 [−17, −12.5]*	−19 [−21, −17]	−20 [−22, −19.5]*	<0.001/<0.001*
Peak GWI (mmHg%)	1,057 [810.5, 1,057]*	1,644 [1,164, 2,558]	2,245 [1,928.5, 2,961]*	0.001/0.02*
Peak GWE (%)	82 [74.5, 86.5]*	88 [82, 90]	89 [86, 93.5]*	0.003/0.001*
Peak GCW (mmHg%)	1,618 [1,153.5, 2,003]*	2,387 [1,818, 2,745]	2,585 [2,262.5, 3,262]*	0.004/0.02*
Peak GWW (mmHg%)	355 [235, 489]	329 [164, 520]	191 [113, 299.5]	0.057

CAD, coronary artery disease; CKD, chronic kidney disease; DM, diabetes mellitus; DSE, dobutamine stress echocardiogram; GLS, global longitudinal strain; GCW, global myocardial constructive work; GWE, global myocardial work efficiency; GWI, global myocardial work index; GWW, global myocardial wasted work; HR, heart rate; HTN, hypertension; LVEF, left ventricular ejection fraction; SBP, systolic blood pressure; WMSI, wall motion scoring index.

Data are presented as mean ± standard deviation (SD) for normally distributed variables and median [25th–75th interquartile range (IQR)] for non-normally distributed variables. Values marked with an asterisk (^*^) indicate statistically significant differences between the control and significant CAD groups (adjusted *p* < 0.05).

### 3.2 WMSI, GLS and MW analysis

In all patients with abnormal DSE (*n* = 20), peak WMSI (1.3 ± 0.1 vs. 1.2 ± 0.1, *p* = 0.29) did not differ between significant CAD vs. non-significant CAD groups. There was a moderate negative correlation observed between peak WMSI and peak GLS (*r* = −0.58, *p* = 0.001), peak GWI (*r* = −0.44, *p* = 0.002), and peak GWE (*r* = −0.45, *p* = 0.001) in all with abnormal DSE (*n* = 20).

Baseline and peak values for GLS, GWI, GCW, GWW, and GWE are summarized in Table 1. At baseline, MW parameters were comparable across all groups, with no significant differences observed (*p* > 0.05 for all comparisons).

At peak stress, patients with significant CAD had markedly lower peak GLS [−15 (−17, −12.5) vs. −20 (−22, −19.5)%, *p* < 0.001], peak GWI [1,057 (810.5, 1,057) vs. 2,245 (1,928.5, 2,961) mmHg%, *p* = 0.002], peak GWE [82 (74.5, 86.5) vs. 89 (86, 93.5)%, *p* = 0.002], and peak GCW [1,618 (1,153.5, 2,003) vs. 2,585 (2,262.5, 3,262) mmHg%, *p* = 0.003], while peak GWW was higher, but non-significant [355 (235, 489) vs. 191 (113, 299.5) mmHg%, *p* = 0.076] compared to controls. An example of changes in myocardial work parameters in a normal patient without significant CAD (Figures 1A–D) compared to a patient with significant CAD (Figures 1E–H) undergoing DSE is illustrated (Figure 1).

At peak stress patients with non-significant CAD also exhibited a numeric but non-significant decrease in peak GLS [−19 (–21, −17) vs. −20 (−22, −19.5)%, *p* = 0.290], peak GWI [1,644 (1,164, 2,558) vs. 2,245 (1,928.5, 2,961) mmHg%, *p* = 0.121], peak GWE [88 (82, 90) vs. 89 (86, 93.5)%, *p* = 0.496], peak GCW [2,387 (1,818, 2,745) vs. 2,585 (2,262.5, 3,262) mmHg%, *p* = 0.513], and increase in peak GWW [329 (164, 520) vs. 191 (113, 299.5) mmHg%, *p* = 0.461) compared to controls.

**Figure 2:**
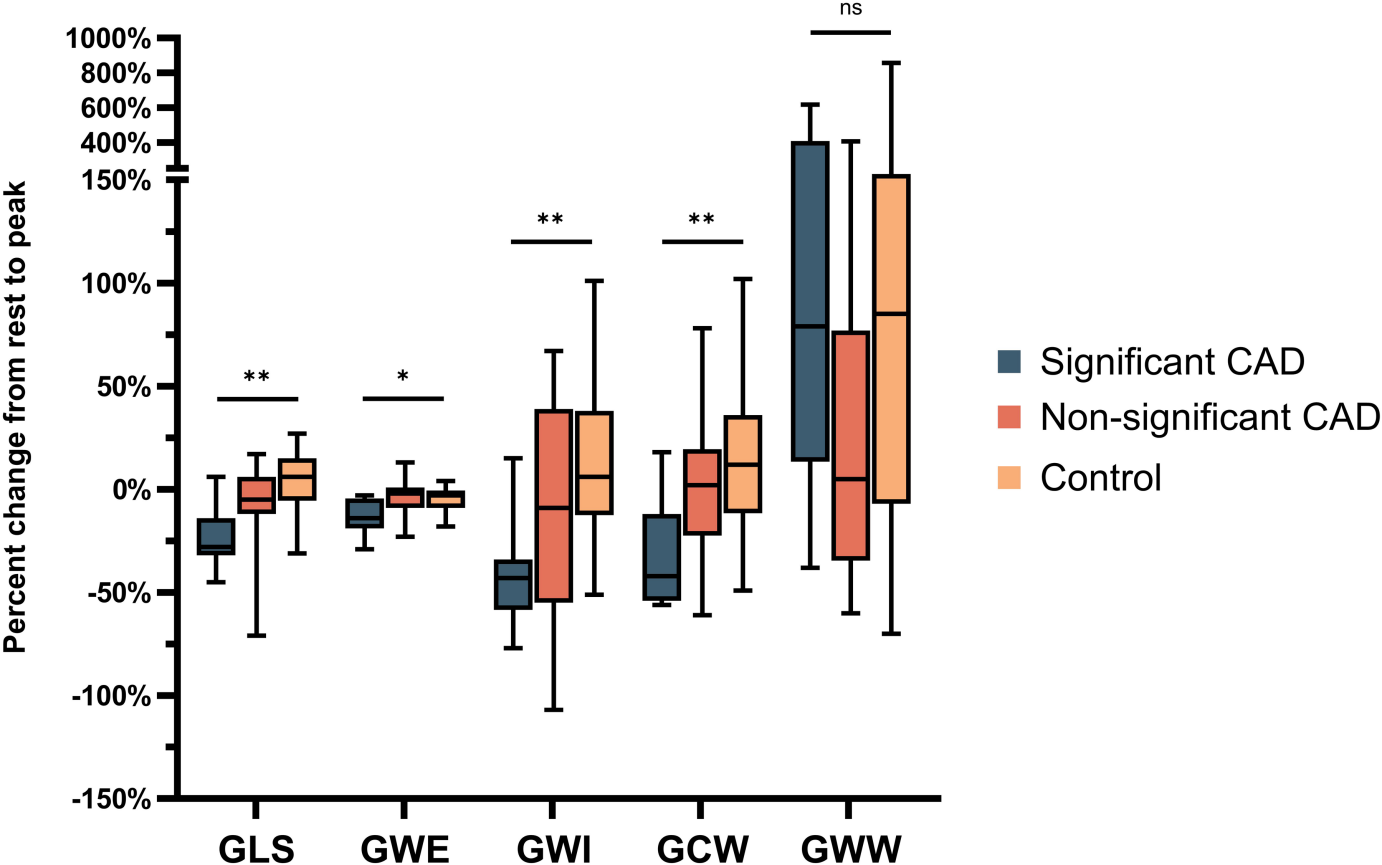
Percent change in GLS, GWE, GWI, GCW, GWW from rest to peak stress undergoing DSE in those with significant and non-significant CAD, as well as controls. CAD, coronary artery disease; GLS, global longitudinal strain; GCW, global myocardial constructive work; GWE, global myocardial work efficiency; GWI, global myocardial work index; GWW, global myocardial wasted work; DSE, dobutamine stress echocardiogram. *p < .05; **p < .01; *ns* = *non-significant*.

In the CAD group, significant percent decrease from baseline to peak in GLS [−28 (–32, −14) vs. + 4 (−5.5, 15)%, *p* = 0.001], GWE [−14 (–19, −4) vs. −3 (−9, −0.5)%, *p* = 0.047], GWI [−58.5 (–43, −34) vs. + 6 (−12.5, 38)%, *p* = 0.002], and GCW [−42 (–54, −12) vs. + 12 (−11.5, 36)%, *p* = 0.004] while there is augmentation of the above parameters in controls (positive sign) (Figure 2). However, the change in GWW was not significant [+79 (13.5, 409) vs. + 85 (–7, 205) %, *p* = 1.0] (Figure 2).

In the non-significant CAD group, there is minimal change from baseline to peak but augmentation of these parameters in control (+sign) albeit non-significant in GLS [−5 (–12, 6) vs. + 4 (−5.5, 15)%, *p* = 0.264], GWE [−2 (–9, 1) vs. −3 (−9, −0.5)%, *p* = 1.0], GWI [−9 (–55, 39) vs. + 6 (−12.5, 38)%, *p* = 0.557], GCW [+2 (−22.5, 19.5) vs. + 12 (−11.5, 36)%, *p* = 1.0], and augmentation GWW in both groups [+5 (−34.5, 77) vs. + 85 (–7, 205)%, *p* = 0.334].

Regional wall motion abnormalities on echo, findings of angiograms, and segments with reduced myocardial work are listed in Table 2. Myocardial work (GWI) tended to be reduced in more segments than the visualized regional wall motion abnormality on DSE and the peak GLS map for ischemia.

**Table 2:** Assessment of regional echocardiographic wall motion abnormality at peak DSE, associated stenotic lesion on coronary angiography, and corresponding myocardial segments with reduced myocardial work index.

Patient #	Stress echo regional wall motion abnormality location	Obstructive lesions on coronary angiography	Myocardial segments with reduced myocardial work index
1	Mid to distal inferior septum, Apex, Inferior wall, WMSI = 1.1	Proximal LAD 80%, Mid RCA 80%	Basal, mid and distal septum. Basal, mild and distal inferior and posterior wall
2	Basal to mid anterior septum, Basal to mid inferior septum, Basal to mid lateral wall, WMSI = 1.38	Ostial LM 50%, Proximal LAD 100%, Proximal RCA 100%, Patent LMA to LAD, SVG to D1 occluded, SVG to OM1 occluded	Basal septum, anterior septum and anterior wall. Basal to mid inferior wall. Basal to mid posterior wall
3	Basal inferior septum, Basal inferior wall, Basal to mid inferolateral wall, WMSI = 1.25	Proximal RCA 99%, Mid RCA 70%, Distal RCA 80%, Mid LM 55%	Basal septum and anterior septum. Basal inferior and posterior wall
4	Distal inferior septum, Distal inferior wall, Anterior septum, Apex, Basal inferolateral wall, WMSI = 1.38	Proximal LAD 75%, Proximal RCA 75%, Distal RCA 25%, Distal LCX 90%	Basal, mild and apical septum. Basal, mid and apical anterior septum
5	Basal inferior septum, Basal Inferior wall, Basal to mid inferolateral wall, WMSI = 1.25	Mid RCA 100% chronic total occlusion	Basal septum, Basal inferior wall
6	Mid to distal inferior septum, Anterior septum, Distal inferior wall, WMSI = 1.5	Ostial LAD 95%, Ostial LCX 90%	Basal to mid septum, anterior septum and anterior wall. Basal to mid lateral wall. Basal to mid inferior and posterior wall
7	Apex, Distal inferior septum, Anterior septum, Anterolateral wall, WMSI = 1.38	Mid LM 30%, Ostial LAD 80%, Proximal LAD to D1 80%	Basal septum and anterior septum. Basal to mid inferior and posterior wall
8	Distal Anterior septum, Apex, WMSI = 1.1	Mid LAD 90%, Distal LCX 80%	Basal, mid and apical septum, anterior septum and anterior wall. Basal, mid and apical inferior wall
9	Basal Inferior septum, Basal inferior wall, Basal to mid inferolateral wall, WMSI = 1.25	Distal LM 95%	Basal to mid septum. Basal anterior wall. Basal lateral wall. Basal to mid inferior and posterior wall

DSE, dobutamine stress echocardiography; D1, first diagonal artery; LAD, left anterior descending artery; LM, left main artery; OM1, first obtuse marginal artery; LCX, left circumflex artery; RCA, right coronary artery; WMSI, wall motion scoring index; SVG, saphenous vein graft.

**Figure 3:**
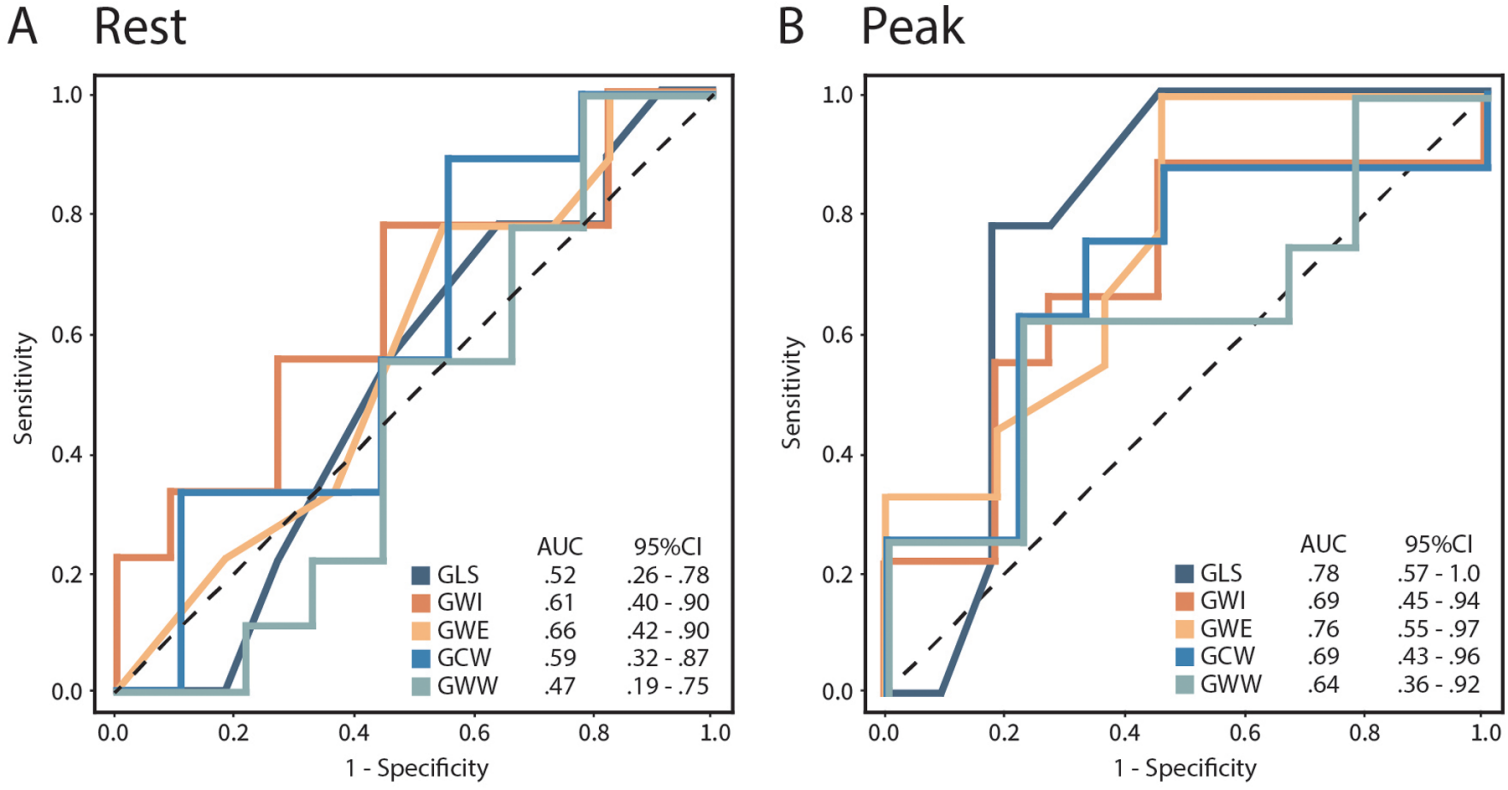
Receiver operating curves (ROC) for rest **(A)** and peak **(B)** GLS, GWI, GWE, GCW, and GWW to detect coronary ischemia in patients with abnormal DSE. *SE*, dobutamine stress echocardiogram; GLS, global longitudinal strain; GWI, global myocardial work index; GWE, global myocardial work efficiency; GCW, global myocardial constructive work; GWW, global myocardial wasted work.

ROC curve analysis of myocardial work parameters showed peak GLS to be the best discriminator for detecting coronary ischemia (AUC 0.78, *p* = 0.01), followed by peak GWE (AUC 0.76, *p* = 0.01) (Figure 3). Although peak GWI did not perform well (AUC 0.69, *p* = 0.17), a 24% drop in GWI at peak stress (AUC 0.74, *p* = 0.07) showed a trend in discriminating coronary ischemia (Figure 3).

Other rest and peak strain-based MW parameters and WMSI were not discriminatory (Table 3). Adding peak GLS > −16.5% + GWE of <87% to an abnormal DSE improved PPV from 45 to 81%.

**Table 3:** Performance of baseline and peak GLS and myocardial work parameters to detect coronary ischemia in with an abnormal DSE and significant CAD.

Echocardiographic variable	AUC (95% CI)	Optimal cutoff (%)	*p*-value
Baseline GLS (%)	0.52 (0.26–078)	−20	0.80
Baseline GWI (mmHg%)	0.65 (0.4–0.9)	1,867	0.27
Baseline GWE (%)	0.66 (0.42–0.9)	90	0.22
Baseline GCW (mmHg%)	0.59 (0.32–0.87)	2,205	0.50
Baseline GWW (mmHg%)	0.47 (0.19–0.75)	98	0.80
Peak GLS (%)	0.78 (0.57–1.01)	−16.5	0.01
Peak GWI (mmHg%)	0.69 (0.45–0.94)	1,602	0.18
Peak GWE (%)	0.76 (0.55–0.97)	86.5	0.01
Peak GCW (mmHg%)	0.69 (0.43–0.96)	1,977	0.17
Peak GWW (mmHg%)	0.64 (0.36–0.92)	351	0.30
% *Δ* GLS	0.67 (0.42–.091)	−2.5	0.19
% *Δ* GWI	0.74 (0.52–0.96)	−24	0.06
% *Δ* GWE	0.67 (0.42–0.9)	−1	0.19
% *Δ* GCW	0.74 (0.48–0.99)	−1	0.09
% *Δ* GWW	0.46 (0.18–0.74)	−156	0.79
Peak WMSI	0.65 (0.31–.038)	1.2	0.31

AUC, area under the curve; DSE, dobutamine stress echocardiogram; GLS, global longitudinal strain; GWI, global myocardial work index; GWE, global myocardial work efficiency; GCW, global myocardial constructive work; GWW, global myocardial wasted work; WMSI, wall motion scoring index; %, percent; *Δ*, change.

### 3.3 Cost analysis

In patients with abnormal DSE and that had non-significant CAD, on secondary testing, overall, 11 tests were performed (6 coronary angiograms, 3 PETs, and 2 SPECTs). The total cost of these tests was $94,443.00, averaging $8,313.00 per patient. If peak GLS and GWE had been employed, 9 secondary tests would have been avoided (5 coronary angiograms, 2 PETs, and 2 SPECTs), resulting in cost savings of $74,466.00. This would result in cost savings of $8,274.00 per patient.

## 4 Discussion

### 4.1 Peak GLS and myocardial work analysis in significant CAD

In our prospective study, we found that peak GLS, GWI, GCW, and GWE obtained at peak stress DSE decreased in patients with abnormal DSE and significant CAD and increased significantly in controls. On the contrary, these parameters did not change appreciably in patients with abnormal DSE and non-significant CAD. Peak GWE, and 24% decrease in GWI at peak showed a trend in discriminating coronary ischemia, and regional MW index was reduced in more segments than regional wall motion abnormality on abnormal DSE.

Similar findings were previously reported by several authors (20, 21). Borrie et al. (20) reported a decrease in GWI in segments identified as ischemic at the peak of exercise. While in some cases, these findings were reflective of the presence of significant CAD, in others no significant CAD was found. In our study, peak myocardial work efficiency performed well in its discriminatory power (AUC 0.76, *p* = 0.01). This was evident by a small decrease in the peak GCW (32%) and a marked increase in peak GWW (97%). We concurred that in patients with significant CAD, the ischemic response is mediated by a minimal decrease in constructive work and a greater increase in wasted work. Peak GLS was discriminatory in detecting coronary ischemia [AUC 0.78, 95% CI (0.57,1.0), *p* = 0.01], also shown by others (15, 20) but with a lower AUC, and was not predictive of coronary ischemia a multivariate model. Even though GLS was discriminatory in our cohort, it has a wide confidence interval. We suspect that this wide range may be a result of higher burden of multivessel disease compared to Halibi et al. (44% vs. 20%) (21). These findings should be validated in a larger cohort.

Borrie et al. (20) also reported that MW tended to be reduced in more segments than the visualized regional wall motion abnormality and the peak GLS map for ischemia. This was also seen in our study. The cause of this cannot be elucidated accurately but may be related to the subjective threshold for change of color on the myocardial work map. Similarly, ischemia in the apical cap by peak GLS was not seen in 2 cases (Table 2, case no. 4 and 8), despite apical hypokinesis and concurrent angiographic stenosis of the mid to distal LAD (left anterior descending artery). The presence of a base-to-apex GLS gradient (22), that is augmented at peak SE, may be well below the threshold for color change on the GLS map identifying ischemia at peak stress.

The usefulness of peak MW strain parameters was previously described by Lin et al. (15) in patients referred for treadmill stress echocardiography showing peak GLS and peak GWE outperforming peak wall motion in detecting coronary ischemia. We performed a similar study with the addition of assessing the additive value of the above parameters to standard DSE in detecting coronary ischemia. We decided to study patients undergoing DSE since GLS is angle-independent and is less susceptible to translational motion and tethering, which allows for better acquisition of DSE images compared to treadmill stress echocardiography. Despite a smaller cohort of our patients, we believe the results of GLS, and strain-derived MW parameters were comparable to other studies. However, the absolute values of peak MW and peak GLS in the significant CAD group were lower than reported by others (22, 23).

Resting GLS, GWI, and GCW have been shown to be reduced, and GWW to be increased, at rest in patients with normal LVEF and no regional wall motion abnormalities yet found to have multivessel CAD (14), with a resting GWI threshold of 1,810 mmHg%, indicative of the presence of significant CAD. These changes were attributed to reduced resting myocardial efficiency in patients with significant CAD. In our study, all patients had normal wall motion and LVEF at rest. Despite multivessel disease being present in most of the significant CAD group, resting GWI was above the reported threshold. Our findings were consistent with other studies that investigated resting strain and myocardial work efficiency in healthy individuals and patients with cardiovascular diseases (24, 25). Al Mahdiui et al. (25) reported normal values of these parameters in healthy patients with a median threshold for myocardial efficiency of 96%. They found similar values of myocardial efficiency in patients with risk factors for CAD. Decreased resting myocardial efficiency was only noted in patients with structurally abnormal hearts (prior myocardial infarction and/or heart failure with reduced systolic function) (25).

In our controls, peak GWW [191 (113, 299.5) vs. 79 mmHg%], peak GWI [2,245 (1,928.5, 2,961) vs. 1,896 ± 308 mmHg%], peak GCW [2,585 (2,262.5, 3,262) vs. 2,232 ± 331 mmHg%] were higher, while peak GWE [89 (86, 93.5) vs. 96%] was lower compared to values reported in healthy individuals (11). This discrepancy may be attributed to the higher prevalence of hypertension in our control group, which likely contributed to increased afterload and altered myocardial work indices.

### 4.2 Utility of myocardial work parameters in hypertensive response to DSE

Patients with a hypertensive blood pressure response during DSE are more likely to have stress-induced myocardial ischemia compared to patients with normal BP response (26). While they may have CAD, the presence of severe CAD (>70% luminal stenosis) is less likely. Abnormal DSE results were more commonly encountered in older females with a history of DM, baseline hypertension, and preserved baseline LV function. A discordant relationship between the WMSI at peak exercise and the extent and severity of CAD was also noted (26). Our abnormal DSE non-significant CAD group (*n* = 11) were also older hypertensive and diabetic females with highest BP response to DSE (although statistically non-significant). We found discordant results between GLS and GWI in these patients, suggesting that load-dependent factors may have influenced their stress echocardiography results.

While GLS has proven useful in assessing LV function, its reliance on load conditions remains a key limitation. Recent studies have demonstrated that MW analysis, which integrates LV pressure-strain relationships, provides a more load-independent assessment of myocardial performance (27). This may explain why GWE, enhances the positive predictive value of DSE, refining its diagnostic accuracy and reducing unnecessary secondary testing. By incorporating MW parameters into conventional DSE interpretation, we were able to account for the influence of loading conditions, leading to better differentiation between patients with significant and non-significant CAD.

**Figure 4:**
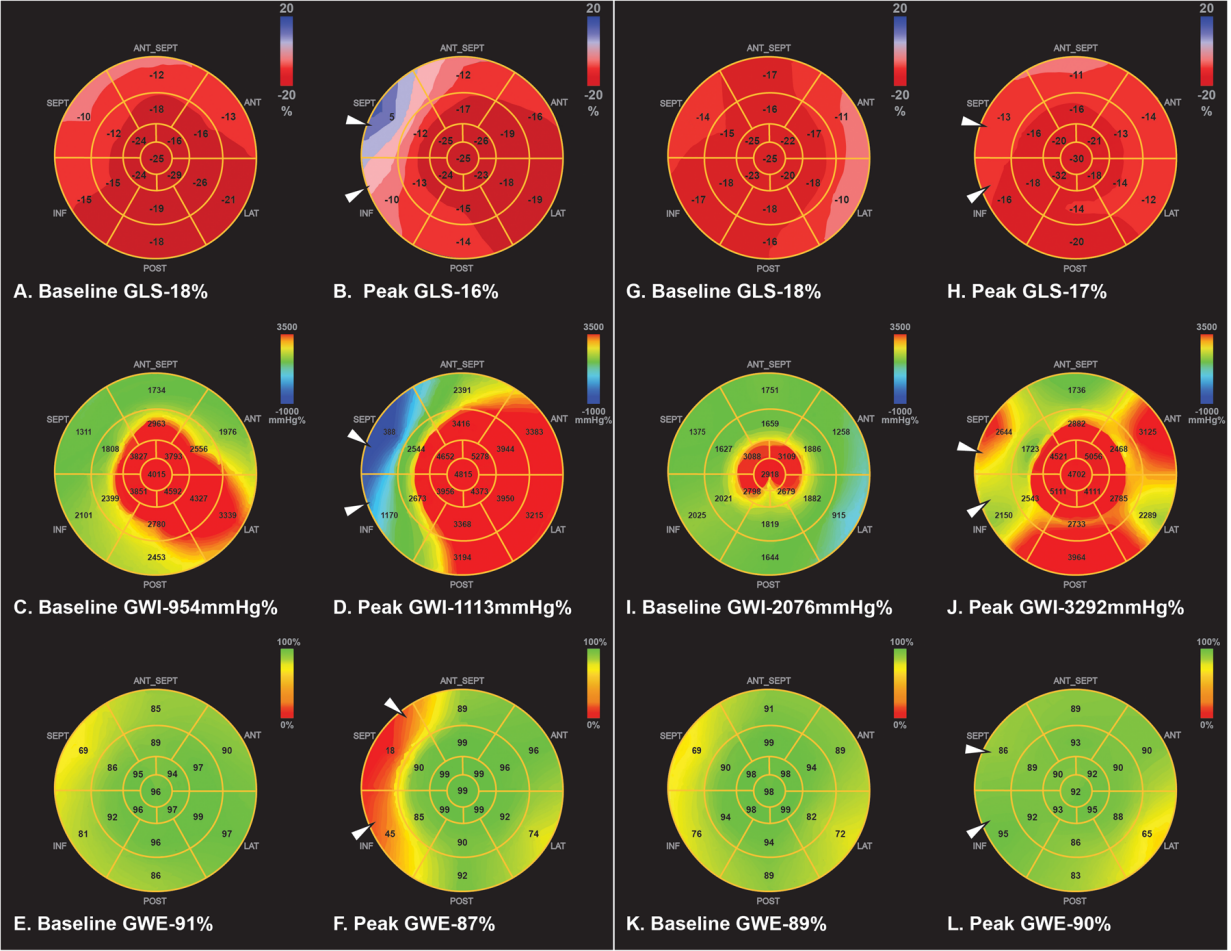
Example of two patients with hypertensive response during DSE. First patient was a 56 y/o female that had basal inferior and inferior septum wall motion abnormality at peak stress. Peak SBP was 262/78 mmHg. GLS and regional longitudinal strain were decreased at peak stress in the RCA territory compared to rest **(A,B)**. GWI was increased from rest to peak stress, but regional MW was deceased in the RCA territory **(C,D)**. Peak GWE and regional MW efficiency was also decreased in the RCA territory **(E,F)**. Cardiac catheterization showed total occlusion of the mid RCA with collaterals to the distal RCA. Second patient was a 41 y/o male that showed similar wall motion abnormality (basal inferior and inferior septum). Peak SBP was 250/111 mmHg. However, peak GLS and regionals longitudinal strain were unchanged **(G,H)**. GWI and regional MW in the RCA region were increased at peak stress **(I,J)**. GWE and regional MW was preserved at peak stress **(K,L)**. Subsequent cardiac PET was negative for coronary ischemia. DSE, dobutamine stress echocardiogram; GLS, global longitudinal strain; GWI, global myocardial work index; GWE, global myocardial work efficiency; MW, myocardial work; RCA, right coronary artery; PET, positron emission tomography.

To illustrate this, we show rest and peak myocardial strain parameters in two patients (Figure 4). Both had hypertensive blood pressure responses (peak SBP >182 mmHg) and wall motion abnormality in the right coronary artery (RCA) distribution. Patients with significant CAD had decreases in peak GLS, GWI, and GWE in the RCA territory (Figures 4A–F), while patients with non- significant CAD had a slight decrease in peak GLS, augmentation of GWI, unchanged GWE, and no decrease in the above parameters in the RCA territory (Figures 4G–L).

### 4.3 Implications of cost savings

Finally, cost savings in value-based health care is advantageous (28). The cardiovascular costs of care are rising, with $2 out of every $10 being spent on outpatient visits and diagnostic testing (29). We demonstrated that applying peak GLS and GWE to conventional DSE leads to cost savings from avoiding downstream testing. Reducing unnecessary testing can result in a sizeable economic impact (30).

While GLS and GWE improve the PPV of DSE, they do not eliminate the need for secondary testing. The potential impact of false negatives—patients with normal GLS and GWE but significant CAD—must be considered, as undiagnosed CAD may lead to repeat testing, emergency visits, or delayed interventions, increasing downstream costs. The American College of Cardiology (ACC) and American Heart Association (AHA) 2021 chest pain guidelines emphasize that inconclusive or false-negative noninvasive test results contribute to higher healthcare costs by prompting additional testing (31). Similarly, the PROMISE trial found that patients with inconclusive noninvasive tests were more likely to undergo further testing and incur higher medical expenses than those with conclusive negative results (32).

Future studies should validate these cut-off values in larger, prospective cohorts and incorporate a broader cost-effectiveness analysis that considers both cost savings from reducing unnecessary tests and the economic burden of missed diagnoses.

### 4.4 Limitations

The results of our small study are proof of concept and should be validated in a larger population. In this study, we reported a 15% exclusion rate, which is comparable to others (21, 24). The rate of false-positive studies were higher in our cohort than reported by others (26), but this likely represents high-volume non-academic echocardiography laboratories, where the application of our results would be even more meaningful. Univariate or multivariate logistic regression to ascertain independent predictors of coronary ischemia was not performed due to the small sample size.

The degree of coronary artery stenosis was assessed visually, which limits the diagnostic accuracy of physiologically significant stenosis. There is a low possibility of false-negative SPECTs, which could have affected PPV calculation. Finally, the lack of confirmatory tests in the control patients precluded the calculation of specificity of MW parameters. The results of our study may not apply to other populations.

MW parameters were only assessed at baseline and peak DSE. Its assessment at low dose and during recovery may have resulted in a more accurate assessment. Finally, the data was acquired at 50–80 fps during exercise, which is lower than recommended (24), but is comparable with other studies (15, 23).

## 5 Conclusion

Adding peak global longitudinal strain and peak myocardial work efficiency to conventional Dobutamine stress echocardiographic analysis of wall motion improved its diagnostic accuracy. This improved accuracy may result in cost savings.

## Data Availability

The original contributions presented in the study are included in the article/supplementary material, further inquiries can be directed to the corresponding author.
